# Optimizing Benefits‐Harms of *H. pylori* Screen‐and‐Treat Programs Tailored to the Regional Settings

**DOI:** 10.1111/hel.70111

**Published:** 2026-03-04

**Authors:** Duco T. Mülder, Yi‐Chia Lee, Mario Dinis‐Ribeiro, Melissa McLeod, Jin Young Park, Iris Lansdorp‐Vogelaar

**Affiliations:** ^1^ Early Detection, Prevention and Infections Branch International Agency for Research on Cancer Lyon France; ^2^ Department of Public Health Erasmus Medical Center Rotterdam the Netherlands; ^3^ Department of Internal Medicine, College of Medicine, National Taiwan University and Departments of Medical Research and Internal Medicine National Taiwan University Hospital Taipei Taiwan; ^4^ Department of Gastroenterology, Porto Comprehensive Cancer Center and RISE@CI‐IPO (Health Research Network), 4200‐072 Porto, Portugal & MEDCIDS (Department of Community Medicine, Health Information, and Decision), Faculty of Medicine University of Porto Porto Portugal; ^5^ Department of Public Health University of Otago Wellington New Zealand

## Abstract

This article outlines how decision modeling can be used to optimize the cost‐effectiveness of *H. pylori* screen‐and‐treat programs. Decision models enable the translation of data from pilot studies into locally tailored strategies by adapting test modalities, treatment options, and the need to retest specific to the local setting. We summarize existing evidence from modeling studies, which consistently demonstrate that *H. pylori* screen‐and‐treat is cost‐effective across diverse populations. In addition, we discuss how decision modeling can support resource allocation, promote health equity, and guide implementation planning. Integrating *H. pylori* screen‐and‐treat into established preventive programs, such as colorectal cancer screening, may further increase efficiency and feasibility. The article concludes with a proposed research agenda to advance efficient *H. pylori* screen‐and‐treat programs across the globe.

## 1 Introduction

The articles in this special issue highlight the variety of gastric cancer prevention strategies implemented worldwide, with a strong emphasis on population‐based *Helicobacter pylori (H. pylori)* screen‐and‐treat programs. Collectively, these articles demonstrate the potential of an *H. pylori* screen‐and‐treat approach to reduce the burden of gastric cancer. However, translating success strategies to other settings can be challenging, as the optimal strategy depends on local circumstances, such as the disease burden, resource availability, and the structure of the healthcare system.

This paper aims to support health policymakers in designing efficient *H. pylori* screen‐and‐treat programs tailored to their context. The article starts by delving into the role of decision modeling in the optimal implementation of *H. pylori* screen‐and‐treat and what information needs to be collected to allow effective decision modeling for the local context. Notwithstanding the need for local evidence, we then discuss currently available international evidence with respect to the cost‐effectiveness of *H. pylori* screen‐and‐treat, and optimal strategies for implementation (e.g., which test, what age, etc.). Furthermore, we explain the potential synergy when combining *H. pylori* screen‐and‐treat with existing preventive interventions.

The content of this article provides an update of the findings in chapter 9 of the working group report on population‐based *H. pylori* screen‐and‐treat strategies for gastric cancer prevention [1]. This report, written by an international interdisciplinary panel convened by the International Agency for Research on Cancer (IARC), aims to provide guidance on program implementation. The authors of this article were part of the working group and participated in the expert meeting in February 2025. Since the publication of the original report, new evidence and modeling studies have emerged warranting an updated synthesis of the literature. We therefore updated the literature search to include all published articles until December 2025. Furthermore, this article extends the original report by exploring how decision modeling can inform *H. pylori* screen‐and‐treat programs beyond cost‐effectiveness, such as applications in healthcare resource planning and addressing health disparities.

## 2 Decision Modeling for Regional *H. pylori* Screen‐And‐Treat Optimization

Although the optimal strategy for *H. pylori* screen‐and‐treat depends on local conditions, it is not feasible to perform local clinical studies addressing all variables and dimensions of the program. Alternatively, decision modeling can translate findings of clinical studies to the local settings in a different way to estimate whether *H. pylori* screen‐and‐treat provides good value for money in the local setting and under what conditions.

Decision modeling is a structured process that predicts the outcomes of certain scenarios, offering valuable insights to policy makers and stakeholders. This may help in both the implementation and evaluation phase of preventive interventions.

Similarly, decision modeling should be used in the decision‐making phase of *H. pylori* screen‐and‐treat programs to establish for the local setting whether its benefits outweigh its harms and whether the required resources are economically balanced with the net benefits. If a positive decision on implementation has been made, decision models can next be used in the preparatory phase before implementation to estimate annual resource requirements for laboratory testing, drug availability, endoscopic follow‐up capacity, etc. to help planning of the *H. pylori* screen‐and‐treat program. Different roll‐out schedules can be compared to best accommodate resource constraints, and potential bottlenecks in implementation can be identified and tackled where necessary. After that, during implementation, decision modeling can be used to compare program outcomes (and their distribution over population subgroups) with expectations based on prior modeling and/or pilot studies. Moreover, decision modeling can evaluate how best to adjust the program if it does not perform according to expectations. Finally, modeling can be used to make predictions of the long‐term benefits of the program. This is especially important in the light of the long lag time between the implementation of an *H. pylori* screen‐and‐treat strategy and its actual reduction in gastric cancer incidence and mortality.

## 3 Data Requirements for Regional Modeling

The aim of decision modeling is to extrapolate findings of clinical studies to different settings and strategies. However, for valid extrapolation to the local setting, two requirements need to be met: (1) evidence for the effectiveness of screening is available for settings comparable to the local situation; and (2) good‐quality data is available to inform model parameters to the local setting. Currently, most trial evidence relies on the long‐term benefits of *H. pylori* screen‐and‐treat, which comes from studies in Asia; long‐term model results for the non‐Asian context should, therefore, be interpreted with caution.

With respect to data availability, access to more elaborate and detailed data enables more precise estimations. Data requirements can generally be categorized into three main groups: demographic data, disease & testing data and outcome data (Table 1).

**TABLE 1:** General data requirements for cost‐effectiveness modeling.

Demographic^a^	Disease and testing^a^	Outcome
Birth tables	*H. pylori* prevalence by age	Costs of and associated to the test and procedure
Life‐tables (life‐expectancy)	Gastric cancer incidence by localization (cardia vs. non‐cardia) and histology (Lauren‐classification) by age	Treatment costs (stage specific, ideally split by phase of care)
	Observed cancer stage distribution	Estimates of disutility per test procedure^b^
	Stage‐specific cancer survival	Stage‐specific estimates of disutility to gastric cancer
	Test participation in pilot studies (initial participation and treatment adherence)	

^a^ Data should be reported stratified by variables of interest such as gender, geographic region, socioeconomic status or migration history.

^b^ Disutility in the context of a *H. pylori* screen‐and‐treat refers to individual's negative aspects of the screening strategy, such as physical discomfort related to antibiotic treatment and mental distress about the cancer risk [2]. If unavailable, proxies based on existing literature could be considered for use in decision modeling.

Developing and calibrating decision models is a complex and time‐consuming task that requires specialized expertise in statistical modeling and epidemiology. Instead of developing independent decision models, health policymakers may choose to collaborate with established modeling consortia such as the Cancer Intervention Screening Network (CISNET) [3] or Decision Analysis in R for Technologies in Health (DARTH) group [4].

## 4 Current Evidence from Decision Modeling on *H. pylori* Screen‐And‐Treat Strategies

Decision models should always reflect the local demographic and epidemiological data to inform health policy. While such context‐specific studies are needed to maximize the benefits of *H. pylori* screen‐and‐treat, some lessons can be learnt from existing decision modeling studies. Especially when results are found to be robust across different *H. pylori* prevalence and gastric cancer risk settings, it is likely that these results are generalizable to the local setting. Even though decision models can serve multiple purposes, such as evaluating the impact of interventions on healthcare resources or equity, most applications of *H. pylori* screen‐and‐treat modeling have focused on cost‐effectiveness. The discussion of current evidence will therefore center on that aspect.

## 5 Evidence for the Cost‐Effectiveness of *H. pylori* Screen‐And‐Treat

Four reviews have assessed the cost‐effectiveness of *H. pylori* screen‐and‐treat [5, 6, 7, 8]. Three reviews included studies from countries all over the world, with different levels of *H. pylori* prevalence and gastric cancer burden [6, 7, 8]. One review specifically focused on cost‐effectiveness in Western countries, with a lower burden of *H. pylori* and gastric cancer [5]. All four reviews concluded that a strategy of *H. pylori* screen‐and‐treat is cost‐effective in reducing gastric cancer incidence and mortality. Of all 18 studies included in the reviews, only two found *H. pylori* screen‐and‐treat to result in net cost‐savings compared to a situation without testing. In these studies, cost‐savings from preventing dyspepsia were also considered in addition to those of gastric cancer prevention.

The literature was again reviewed for the IARC working group report, finding an additional five studies [1]. Interestingly, four of these studies found that *H. pylori* screen‐and‐treat not only gained life years (LY) by preventing gastric cancer but was also cost‐saving to the health system (compared to no testing) [9, 10, 11, 12]. In the fifth study, *H. pylori* screen‐and‐treat was not found to save costs, but still resulted in a favorable balance between the additional costs and benefits compared to no testing [13].

The search query (Supporting Information) was again updated for this study, yielding an additional five studies all conducted in Asian countries with a high gastric cancer risk (Table 2). Four out of the five studies found *H. pylori* screen‐and‐treat to be cost‐saving [11, 14, 16, 17]. The fifth study found *H. pylori* screen‐and‐treat to be efficient, yet concluded that risk‐based endoscopy screening was the optimal strategy [15].

**TABLE 2:** Overview of studies on the cost‐effectiveness of *H. pylori* screen‐and‐treat published between 2023 and 2025.

Author, year	Country	Population simulated	Strategies evaluated	Test characteristics	Test costs	Costs per QALY/LY
Nakatani, 2023 [14]	Japan	40 and 50‐year old employees	Adding HPA test to UGI	HPA sensitivity: 93% HPA specificity: 79%	HPA: $9.1	cost‐saving
Zheng, 2023 [15]	Hypothetical high‐risk country	> 40 years old	14C UBT and NGCS risk‐based screening	14C UBT sensitivity: 96% 14C UBT specificity: 93%	UBT: $25.56	$76.17 UBT compared to no‐screen $5492.84 UBT compared to NGCS
Kowada, 2024 [11]	Japan	15‐year old cohort	HPA. If positive, UBT. At ages 15, 18, 20, 30, …, 80	HPA sensitivity: 93% HPA specificity: 99% UBT sensitivity: 96% UBT specificity: 93%	HPA: $16.27 UBT: $36.90	Cost‐saving at ages 15–40 and age 80. At ages 50–70: $2476–2479
Ma, 2025 [16]	China	Families ≥ 2 people	Random testing and if positive, test family members vs. Random testing. All 13C‐UBT	UBT sensitivity: 95.5% UBT specificity: 94.7%	UBT: $24.28	Family‐based: cost‐saving
Li, 2025 [17]	China	Cohort of 20‐year olds	UBT at ages 20, 30, 40. One‐time test + every 2/3/5 years until age 50	UBT sensitivity: 87.6% UBT specificity: 84.8%	UBT: $11.63	All one‐time tests cost‐saving. Costs compared to no‐screen of repeat tests every 2/3/5 years: At age 20: $215/$122/$47 At age 30: $201/$81/$15 At age 40: $98/cost‐saving/cost‐saving

**FIGURE 1:**
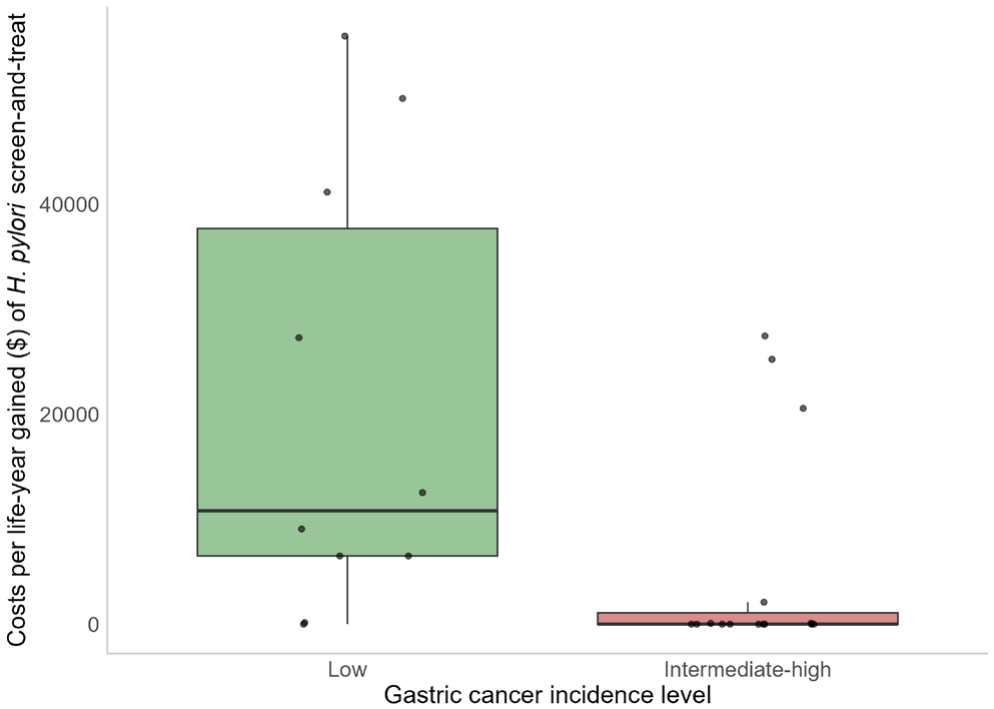
Costs per life‐year gained for *H. pylori* screen‐and‐treat as observed in reviewed cost‐effectiveness analyses, stratified by gastric cancer incidence level. Boxes show the interquartile range, horizontal lines indicate the median, and points represent individual studies. Costs per life‐year gained have been standardized to present value. Studies demonstrating cost savings are set at 0. As willingness‐to‐pay thresholds and price levels differ across settings, the interpretation of costs per life year gained may differ among studies and settings. Data based on Lansdorp‐Vogelaar et al. [1] combined with the extra studies extracted in this study (Table 2).

Gastric cancer burden has an important influence on the cost‐effectiveness of *H. pylori* screen‐and‐treat programs. With a higher burden of disease, more deaths can be prevented with the same number of tests, resulting in a more favorable balance between benefits and resources required than in lower incidence settings. Although *H. pylori* screen‐and‐treat was found to be cost‐effective across all settings, the costs per life‐year gained were therefore typically lower in studies performed in high‐risk areas (Figure 1). One study explicitly studied the impact of *H. pylori* prevalence and gastric cancer burden on the cost‐effectiveness of *H. pylori* screen‐and‐treat [18]. This study concluded that in countries with intermediate to high (in this study ASR ≥ 17 per 100,000 person‐years) gastric cancer incidence, *H. pylori* screen‐and‐treat would be cost‐saving. However, the study also showed that even in low‐incidence countries (in this study: ASR 6 per 100,000 person‐years) *H. pylori* screen‐and‐treat resulted in a favorable balance between costs and health benefits.

It is important to note that a formal quality assessment of the included decision modeling studies has not been performed in any of the reviews, nor have we engaged in such an endeavor ourselves. Nevertheless, the consistency of the finding that *H. pylori* screen‐and‐treat is cost‐effective across studies provides additional confidence in the validity and robustness of this finding.

## 6 Optimizing the Cost‐Effectiveness of *H. pylori* Screen‐And‐Treat Strategies for Gastric Cancer Prevention

When *H. pylori* screen‐and‐treat is implemented, decisions need to be made on the test modality, screening age (s), which drugs to use for eradication, whether to perform confirmation of eradication testing, and whether to perform resistance testing prior to eradication. In this paragraph, we summarize the current available evidence on which strategies provide most value for money.

### 6.1 Test Modality

Only three studies directly compared Carbon Urea Breath Test (^13^C‐UBT) with serology testing, with two of them additionally considering Stool Antigen Test (SAT) [9, 19, 20]. In these three studies, a strategy based on ^13^C‐UBT testing was associated with higher costs than serology testing. However, ^13^C‐UBT was also more effective in preventing gastric cancer incidence and mortality and thus gaining more LY. In the study by Yousefi et al. these extra benefits weighed favorably against the extra costs [9]. In the other two studies, the incremental costs per QALY exceeded the considered willingness‐to‐pay threshold, implying that ^13^C‐UBT did not provide good value for money compared to serology in these settings [19, 20]. Two studies comparing SAT to serology and ^13^C‐UBT concluded that SAT was more effective than serology. One of the studies also found SAT to be less expensive than the other test modalities [9] while the other found it to be highly cost‐effective [20].

Another study compared ^13^C‐UBT with polymerase chain reaction (PCR) testing of gastric biopsies and concluded that PCR testing is cost‐effective for gastric cancer prevention [13]. However, testing of gastric biopsies is an invasive strategy and may therefore be less acceptable and scalable than other testing modalities. Moreover, serology and SAT were not considered in this analysis. Consideration of these strategies may have resulted in a less favorable balance between costs and benefits (QALY gained) of PCR testing compared to these strategies.

In conclusion, there is limited evidence on the optimal test for *H. pylori* screen‐and‐treat for gastric cancer prevention with only four decision modeling studies performing direct comparisons between tests. These studies suggest that SAT may be more cost‐effective than serology. However, in general, all tests were found to be cost‐effective for gastric cancer prevention compared to no testing, and none of the tests was consistently dominated in all of the analyses. This finding suggests that the choice of the test may be based on local setting and resource considerations rather than cost‐effectiveness.

### 6.2 Test Age

Eight studies compared the cost‐effectiveness of different ages for *H. pylori* screen‐and‐treat [11, 17, 18, 19, 21, 22, 23, 24]. Three studies concluded that it was optimal to test for *H. pylori* infection at a young age (20 or 30 years), because *H. pylori* testing in cohorts older than that was both less effective and less cost‐effective.^10,2018^ These studies were performed in high‐incidence settings (ASR ≥ 20 per 100,000 person‐years). Another study found that one‐time testing was cost‐effective at all ages 20–40, but that retesting was only cost‐effective at age 40 [17]. The other studies, mostly based in low‐incidence settings (ASR < 10 per 100,000 person‐years) also found *H. pylori* testing to be more effective at these younger ages, but this effectiveness was accompanied by higher costs per life‐year gained. They therefore suggested ages for *H. pylori* testing centered around 40–50 years. These findings suggest that in low‐incidence settings, *H. pylori* screen‐and‐treat might not be cost‐effective before age 40, whereas it may be in high‐incidence settings. However, an important caveat with these findings is that many studies compared different screening ages across different birth cohorts. Given the high correlation between birth cohort and gastric cancer risk, this may indicate that it is more cost‐effective to screen older birth cohorts, rather than older individuals. Although not a cost‐effectiveness study, a model‐analysis within one birth cohort in the United States demonstrated that *H. pylori* screen‐and‐treat had a substantial larger benefit before age 40, reflected by the number‐needed‐to‐treat to prevent one cancer case [25]. Conversely, screening at younger age poses challenges to cost‐effectiveness, as the benefits of cancer prevention will only become apparent further in the future and therefore have a lower present value due to discounting. More studies looking at the optimal age of screening within the same birth cohort are therefore needed to demonstrate whether the increase in efficacy compensates the lack of cost‐effectiveness due to discounting.

### 6.3 Repeat Testing for *H. pylori*

Evidence was limited with respect to repeat *H. pylori* screen‐and‐treat strategies. The purpose of repeated testing might be to account for (re‐)infection or failed eradication therapy. Three studies evaluated repetition of *H. pylori* screen‐and‐treat [17, 18, 22]. The studies considered different intervals (varying from 1 to 10 years) and frequencies (one repeat versus multiple) for repeated testing. Two out of three concluded that the extra benefits of repeat testing did not outweigh the extra resources. A third study suggested that repeat testing could be beneficial in China, although cost per life year gained was higher than for a one‐time test [17]. It is important to point out that evidence on re‐infection rates is scarce, although the rates are estimated to be less than 1% [26]. In the absence of strong evidence, policy makers can best implement a once‐only *H. pylori* screen‐and‐treat strategy. However, pilot studies within these programs, in which a subset of individuals is rescreened after 5–10 years, should be considered to fill this important gap in knowledge and inform future modeling.

### 6.4 Management of *H. pylori* Infection

None of the cost‐effectiveness analyses on *H. pylori* screen‐and‐treat compared different eradication therapy regimens, or the benefit of resistance testing prior to initiating treatment. However, one study each addressed the incremental cost‐effectiveness of confirmation testing of successful eradication [27] and restricting treatment to only those that tested CagA‐positive [28]. Fendrick et al. explicitly compared serological testing with and without confirmatory testing 6 weeks after eradication therapy [27]. Assuming 80% effectiveness of initial eradication therapy, the scenario with the confirmatory test resulted in more life years gained than the strategy without confirmatory testing, but was substantially more costly. This finding suggests that in settings where the eradication rate of initial therapy exceeds 80%, confirmatory testing is not cost‐effective. However, without confirmatory testing in at least a sample of the population it is not possible to establish the *H. pylori* eradication rate.

Harris et al. evaluated the cost‐effectiveness of screening and treating either all *H. pylori* strains or CagA‐positive strains only [28]. Testing and treating only CagA‐positive infection reduced the number treated, the number of cases of anaphylaxis and overall costs of screen‐and‐treat, but it also reduced the number of cancers prevented and life years gained. In all countries for which it was evaluated, the incremental cost‐effectiveness ratio (ICER) for treating all *H. pylori* compared to CagA‐positive strains only was less than $25,100 per life year gained. These results suggest that it is better to screen‐and‐treat for all *H. pylori* rather than CagA‐positive alone.

### 6.5 Family‐Based Testing

A novel screening strategy for *H. pylori* is through family‐based testing, in which family members of positively tested individuals are also tested and treated for infection. This strategy started with a Chinese consensus report [29] and has now been adopted by several clinical guidelines [30, 31, 32]. From a cost‐effectiveness perspective, Ma et al. demonstrated that family‐based *H. pylori* screen‐and‐treat is a cost‐saving strategy and provides better value for money than traditional screen‐and‐treat in China. Further studies on family‐based testing are needed to confirm its effectiveness in different settings.

## 7 Decision Modeling for Resource Planning

In addition to cost‐effectiveness, feasibility and successful implementation depend on access to healthcare facilities, availability of trained personnel, and follow‐up care. Decision analysis not only measures costs and benefits, but also the intermediate aspects of the screening process, such as the number *H. pylori* tests needed, the number of antibiotic treatments needed, hospital visits, etc. This information helps policymakers prepare to ensure availability of resources and health professionals that are adequately trained to perform their role in the *H. pylori* screen‐and‐treat program. Such information is especially important in light of recent shortages of healthcare personnel and antibiotics [33].

Decision modeling can be used to project the required resources to implement a strategy. An illustrative example is how the MISCAN‐Colon model affected resource planning during the roll‐out of the successful Dutch colorectal cancer (CRC) screening program. In the implementation phase, the chosen FIT resulted in longer waiting lists for colonoscopy than expected. MISCAN‐Colon was considered to address the capacity constraints while maximizing screening benefits. The model analysis demonstrated that adjusting the cut‐off, rather than prolonging test intervals or changing eligible screening ages, was the most effective way to ease colonoscopy demand [34, 35].

The availability of resources and needs for *H. pylori* screen‐and‐treat programs may differ substantially between low‐and‐middle‐income countries (LMICs) and high‐income countries (HICs). While LMICs may face a higher disease burden and *H. pylori* prevalence, the lower level of available resources is often reflected in a lower willingness‐to‐pay threshold in CEA. This implies that the costs per life‐year gained need to be lower for the intervention to be considered cost‐effective. In addition to financial constraints, access to tests and antibiotic treatments may be limited in LMICs. Consequently, recommendations developed for HICs may not be feasible in LMICs. Decision modeling can help bridge this gap by integrating evidence with local data to identify the most efficient use of available resources. For instance, in breast cancer screening, modeling demonstrated that starting screening at later ages in LMICs may maximize benefits if screening resources are scarcer than in HICs [36].

## 8 Addressing Health Disparities

Decision modeling can also be applied to address health disparities. While traditional CEA often focuses on cost‐effectiveness as the primary output, there is a growing body of literature on CEA methods that can additionally consider the distributional and equity impacts of interventions [37, 38]. Two key examples of methods are the distributional cost effectiveness analysis (DCEA) and the extended cost effectiveness (ECEA) analysis. DCEA involves modeling an intervention by population subgroup, incorporating a measure of opportunity cost and then using relative and absolute measures of inequality to identify the service configuration that maximizes health while also minimizing ‘unfair’ health inequality [39]. ECEA is an approach that has been developed to address equity concerns relating to medical impoverishment in low‐ and middle‐income countries, where the majority of health care is funded through out‐of‐pocket payments [40, 41].

One study based in New Zealand examined a screen and treat program targeted by age, sex and ethnicity. This study found that while a population‐wide program would not be cost‐effective, targeting the program to Indigenous Māori (who have a higher burden of *H. pylori* and gastric cancer) would be cost‐effective [42].

## 9 Combinations with Colorectal Cancer Screening Programs to Create Synergy

A ‘one‐stop‐shop’ approach combining *H. pylori* screen‐and‐treat with other healthcare interventions may provide synergistic benefits.

One potential synergistic approach for *H. pylori* screen‐and‐treat is the combination with CRC screening. Many CRC screening programs across the world are based on non‐invasive collection of feces, which would combine well with SAT. The feasibility of a combined approach has been established both in Asia [43] and Europe [44]. Nieuwenburg et al. demonstrated that *H. pylori* antigen measurement can be performed in FIT stool samples with similar test performance compared to standard SAT [44]. Since the FIT is widely used in clinical practice, this approach may conveniently enable dual prevention of cancer in both the upper and lower gastrointestinal tracts.

Lee et al. combined the SAT with FIT in the screening of both upper and lower gastrointestinal lesions in a population with high prevalence of digestive tract diseases [43]. They compared three scenarios in a hospital cohort: the usage of SAT in all individuals; only among those with negative FIT; or only among those with negative colonoscopy. The SAT and FIT were therefore used as primary tests for the presence of upper and lower gastrointestinal lesions. The sensitivity of SAT for detecting gastric cancer did not differ and was approximately 50%. Importantly, three‐quarters of gastric cancers were diagnosed as stage I‐II disease. In the same study, but within a validation community cohort, the positive predictive value for upper gastrointestinal lesions using SAT was approximately 32%.\n.

Moreover, a randomized clinical trial inviting approximately 150,000 people to participate in either SAT plus FIT or FIT alone demonstrated an increased participation rate of about 14% of FIT combined with SAT compared with FIT alone [45]. This implies that combined screening attracts a larger proportion of individuals to engage in the screening program. Therefore, utilizing the existing FIT screening framework may be advantageous as outlined in Table 3.

**TABLE 3:** Potential advantages of using the FIT program as the foundation for offering screening and treatment for *H. pylori* infection for gastric cancer prevention.

Eligibility	The eligibility criteria for FIT are shifting towards younger ages, at which *H. pylori* treatment is considered to be of greater benefits.
Invitation	Stool sample‐based tests are more acceptable and accessible for people compared to invasive procedures like endoscopies
Participation	The participation rate of FIT may be increased by adding *H. pylori* stool antigen tests
Testing	Both tests use stool samples, making it easy to distribute them together
Management	The management of *H. pylori* infection has been well established
Cost‐effectiveness	The direct and indirect cost of *H. pylori* testing can be reduced by leveraging the established FIT screening platform

The effectiveness of using the FIT program to offer *H. pylori* screen‐and‐treat depends on the screening age. While most CRC screening programs begin at age 50 [46], the best age to apply *H. pylori* screen‐and‐treat is uncertain. Some studies have suggested that *H. pylori* has most impact before the onset of precursor lesions or in less severe precursor lesions [47]. In that case, it seems likely that the optimal screening age is lower than the start age of CRC screening. Continuation of the current trend towards starting CRC earlier could lead to more potential for synergistic effects in future screening programs. Nevertheless, an intervention at the age of 50 may still be reasonable to achieve meaningful reductions in gastric cancer risk. A randomized clinical trial evaluating the addition of SAT to FIT included participants with a mean age of 58 years [45]. In this trial, an invitation to *H. pylori* screen‐and‐treat reduced gastric cancer incidence by 14% among invited individuals, although the reduction was not statistically significant. However, in post hoc analyses adjusted for non‐adherence to the invitation, a significant 21% reduction in gastric cancer incidence was observed [48]. These analyses should be considered exploratory because of the potential healthy‐screenee bias.

## 10 Pragmatic Trials

The potential benefits of adding *H. pylori* screen‐and‐treat to existing health interventions highlight the need for pragmatic trials and pilot studies, similar to those conducted in Taiwan [48]. Findings from such studies could inform policy decisions and provide key inputs for decision‐analytic models assessing whether *H. pylori* screen‐and‐treat should be implemented as a stand‐alone strategy or integrated within existing infrastructure. Such cost‐effectiveness analysis requires robust evidence on the magnitude of the effectiveness of *H. pylori* eradication at older ages, as well as data on potential cost reductions when tests and treatments are combined with existing screening programs.

## 11 Discussion

This article describes how to optimize the balance between harms and benefits of *H. pylori* screen‐and‐treat. Using decision models, factors such as the choice of test modality, target age and the need for repeat testing, can be tailored to context‐specific factors such as the local disease burden and the available resources. Current evidence from modeling studies was summarized, demonstrating that *H. pylori* screen‐and‐treat was cost‐effective at all investigated levels of gastric cancer incidence. However, the optimal approach differed per setting. We therefore outline practical recommendations for policymakers on the data required to adapt decision models for local use and explore how *H. pylori* screen‐and‐treat can be integrated with colorectal cancer screening.

*H. pylori* infection is known to be the major contributor to gastric cancer [49]. Efforts to combat *H. pylori* should replicate the success seen in other primary prevention programs targeting the elimination of well‐known risk factors, such as human papillomavirus, hepatitis B virus, and hepatitis C virus [50]. Further pilot studies and pragmatic trials are needed to guide the implementation and to inform decision models, such that the optimal strategy can be tailored to the population.

Although more than 23 cost‐effectiveness analyses have been performed on the topic of *H. pylori* screen‐and‐treat, considerable gaps in knowledge and limitations still exist. First, none of the cost‐effectiveness analyses considered the impact of widespread *H. pylori* screen‐and‐treat on antimicrobial resistance (AMR). This has been challenging given that explicit modeling of AMR requires accounting for biological evolution, human behavior and transmission. As standard Markov‐based and microsimulation models are generally unable to capture interaction, integration of cancer natural history models with infectious disease models is needed, similar to approaches adopted for cervical cancer and human papillomavirus (HPV) [51]. Alternatively, the impact of screen‐and‐treat on AMR rates could be estimated indirectly by modeling the projected increases in antibiotic consumption attributable to screen‐and‐treat, and linking these increases to changes in AMR rates. This approach is similar to that presented in the IARC working group report [52]. Second, few cost‐effectiveness studies considered important side benefits of mass *H. pylori* eradication. Such side benefits may include the prevention of peptic ulcer disease, dyspepsia and gastric lymphoma [53]. Associations between *H. pylori* and colorectal cancer have also been established [54, 55]. However, modeling multiple diseases has remained challenging due to the need for separate natural‐history structures, limited data on symptom trajectories and inconsistent definitions across studies. As a result, only two cost‐effectiveness studies considered additional benefits of *H. pylori* screen‐and‐treat related to the prevention of PUD and dyspepsia [56, 57]. The impact of these ancillary benefits and harms on the cost‐effectiveness of *H. pylori* screen‐and‐treat could be considerable. Third, many of the evaluated model analyses rely on literature‐based assumptions regarding disease progression and treatment efficacies. As these factors may evolve over time, ongoing model validation and timely updates are warranted. Finally, few cost‐effectiveness analyses have examined the impact of *H. pylori* screen‐and‐treat programs on health inequalities between subgroups of the target population, such as racial or ethnic minorities or those with lower socioeconomic position.

Most studies included in this article only estimated cost‐effectiveness of one particular strategy for *H. pylori* screen‐and‐treat. The maximum number of strategies considered did not exceed five. However, many questions remain on the most cost‐effective approach to implementing *H. pylori* screen‐and‐treat. This includes questions on which test to use, at what age range to screen, with what frequency, with what treatment regimen to eradicate, and whether to test for resistance prior to treatment or successful eradication after treatment.

Cost‐effectiveness is only one part of the financial question of a screening program, budget impact of the strategy being at least as important. An intervention can be highly cost‐effective or even cost‐saving (better health outcome at lower costs), yet the savings occur later in time and investments are needed now before the start of the program. To date, no studies have been performed to help policy makers gain insight into the annual resource requirements of *H. pylori* screen‐and‐treat. Such decision model studies based on real‐world data from pilot studies are needed for implementation.

New observational evidence and comprehensive decision modeling analyses help address the knowledge gaps identified here. These studies, that capture both the negative and positive side effects of *H*. *pylori* screen‐and‐treat, can provide a final verdict on the balance between benefits, harms and resources of this strategy, evaluate the optimal way to implement and provide policy makers with estimates on what resources are needed now for successful implementation of the program. In Europe, the first step in this direction is being made with the TOGAS project and the Joint Action EUCancScreen. Both projects combine local pilot studies with decision modeling to provide policy makers throughout Europe with the essential information to make informed decisions about *H. pylori* screen‐and‐treat.

## Author Contributions

All authors contributed to the study conceptualization and interpretation of the results. Duco T. Mülder and Iris Lansdorp‐Vogelaar performed the data analysis. Duco T. Mülder drafted the manuscript. All authors critically reviewed the manuscript and approved the final version.

## Funding

The authors have nothing to report.

## Disclosure

Where authors are identified as personnel of the International Agency for Research on Cancer/World Health Organization, the authors alone are responsible for the views expressed in this article and they do not necessarily represent the decisions, policy, or views of the International Agency for Research on Cancer/World Health Organization.

## Conflicts of Interest

The authors declare no conflicts of interest.

## Supporting information


**Data S1:** Supporting Information.

## Data Availability

Data sharing not applicable to this article as no datasets were generated or analysed during the current study.

## References

[1] I. Lansdorp‐Vogelaar , D. T. Mülder , M. Dinis‐Ribeiro , M. McLeod , J. Y. Park , and Y.‐C. Lee , “How to Optimize Cost‐Benefits of *H. Pylori* Screen‐And‐Treat Programs for Gastric Cancer Prevention,” in Population‐Based Helicobacter Pylori Screen‐And‐Treat Strategies for Gastric Cancer Prevention: Guidance on Implementation (IARC Working Group Reports no 12), ed. J. Y. Park (International Agency for Research on Cancer, 2025).

[2] L. Li , J. L. H. Severens , and O. Mandrik , “Disutility Associated With Cancer Screening Programs: A Systematic Review,” PLoS One 14, no. 7 (2019): e0220148. 31339958 10.1371/journal.pone.0220148 PMC6655768

[3] Y. Sereda , F. Alarid‐Escudero , N. A. Bickell , et al., “Approaches to Developing De Novo Cancer Population Models to Examine Questions About Cancer and Race in Bladder, Gastric, and Endometrial Cancer and Multiple Myeloma: The Cancer Intervention and Surveillance Modeling Network Incubator Program,” JNCI Monographs 2023, no. 62 (2023): 219–230. 10.1093/jncimonographs/lgad021 PMC11009510 37947329

[4] F. Alarid‐Escudero , E. M. Krijkamp , P. Pechlivanoglou , et al., “A Need for Change! A Coding Framework for Improving Transparency in Decision Modeling,” PharmacoEconomics 37, no. 11 (2019): 1329–1339. 31549359 10.1007/s40273-019-00837-x PMC6871515

[5] I. Lansdorp‐Vogelaar , R. G. S. Meester , M. Laszkowska , F. A. Escudero , Z. J. Ward , and J. M. Yeh , “Cost‐Effectiveness of Prevention and Early Detection of Gastric Cancer in Western Countries,” Best Practice & Research Clinical Gastroenterology 50 (2021): 101735. 33975689 10.1016/j.bpg.2021.101735

[6] I. Lansdorp‐Vogelaar and L. Sharp , “Cost‐Effectiveness of Screening and Treating *Helicobacter pylori* for Gastric Cancer Prevention,” Best Practice & Research Clinical Gastroenterology 27, no. 6 (2013): 933–947. 24182612 10.1016/j.bpg.2013.09.005 PMC3880867

[7] M. Areia , R. Carvalho , A. T. Cadime , F. Rocha Gonçalves , and M. Dinis‐Ribeiro , “Screening for Gastric Cancer and Surveillance of Premalignant Lesions: A Systematic Review of Cost‐Effectiveness Studies,” Helicobacter 18, no. 5 (2013): 325–337, 10.1111/hel.12050. 23566268

[8] M. Sarmasti , M. Khoshbaten , F. Khalili , and M. Yousefi , “Cost‐Effectiveness of Screening *Helicobacter Pylori* for Gastric Cancer Prevention: A Systematic Review,” Journal of Gastrointestinal Cancer 53 (2022): 1–11. 34694594 10.1007/s12029-021-00726-7

[9] M. Yousefi , S. Rezaei , M. Khoshbaten , and M. Sarmasti , “Cost‐Effectiveness Analysis of Different Screening Strategies for *Helicobacter Pylori* Infection in Iran: A Model‐Based Evaluation,” Helicobacter 28, no. 6 (2023): e13027. 37839058 10.1111/hel.13027

[10] T. Feng , Z. Zheng , J. Xu , P. Cao , S. Gao , and X. Yu , “Cost‐Effectiveness Analysis of the *Helicobacter Pylori* Screening Programme in an Asymptomatic Population in China,” International Journal of Environmental Research and Public Health 19, no. 16 (2022): 9986. 36011621 10.3390/ijerph19169986 PMC9408128

[11] A. Kowada , “Cost‐Effectiveness of Population‐Based *Helicobacter Pylori* Screening With Eradication for Optimal Age of Implementation,” Helicobacter 29, no. 4 (2024): e13120. 39138610 10.1111/hel.13120

[12] Z. Wang , W. Han , F. Xue , et al., “Nationwide Gastric Cancer Prevention in China, 2021–2035: A Decision Analysis on Effect, Affordability and Cost‐Effectiveness Optimisation,” Gut 71, no. 12 (2022): 2391–2400. 35902213 10.1136/gutjnl-2021-325948

[13] A. Oh , H. Truong , J. Kim , S. D. Rustgi , J. A. Abrams , and C. Hur , “Cost‐Effectiveness of Screening With Polymerase Chain Reaction for *Helicobacter Pylori* to Prevent Gastric Cancer and Peptic Ulcers,” Journal of Gastrointestinal Oncology 13, no. 5 (2022): 2186. 36388653 10.21037/jgo-21-911 PMC9660075

[14] M. Nakatani , S. Inoue , and I. Kamae , “Cost‐Effectiveness of Adding a *Helicobacter Pylori* Antibody Test to the Upper Gastrointestinal Series in Gastric Cancer Screening at the Workplace,” Environmental Occupational Health Practice 5, no. 1 (2023): 0010‐OA. 10.1539/eohp.2023-0010-OA PMC11841776 40059928

[15] P. Zheng and J. Liu , “Cost‐Effectiveness Analysis of Hp and New Gastric Cancer Screening Scoring System for Screening and Prevention of Gastric Cancer,” Current Oncology 30, no. 1 (2023): 1132–1145, 10.3390/curroncol30010086. 36661735 PMC9857951

[16] Y. Ma , X. Zhou , Y. Liu , et al., “An Economic Evaluation of Family‐Based Versus Traditional *Helicobacter Pylori* Screen‐and‐Treat Strategy: Based on Real‐World Data and Microsimulation Model,” Helicobacter 29, no. 4 (2024): e13123. 39108224 10.1111/hel.13123

[17] Y. Li , S. Zhu , Y. Liu , D. He , Y. Liu , and H. Li , “Economic Evaluation of Preventing Gastric Cancer by Eliminating *Helicobacter pylori* Infection in China,” Scandinavian Journal of Gastroenterology 60, no. 4 (2025): 327–335. 40035736 10.1080/00365521.2025.2473020

[18] J. M. Yeh , K. M. Kuntz , M. Ezzati , and S. J. Goldie , “Exploring the Cost‐Effectiveness of *Helicobacter pylori* Screening to Prevent Gastric Cancer in China in Anticipation of Clinical Trial Results,” International Journal of Cancer 124, no. 1 (2009): 157–166. 18823009 10.1002/ijc.23864 PMC2597699

[19] F. Xie , N. Luo , G. Blackhouse , R. Goeree , and H.‐P. Lee , “Cost‐Effectiveness Analysis of *Helicobacter pylori* Screening in Prevention of Gastric Cancer in Chinese,” International Journal of Technology Assessment in Health Care 24, no. 1 (2008): 87–95. 18218173 10.1017/S0266462307080117

[20] F. Xie , D. O'Reilly , I. L. Ferrusi , et al., “Illustrating Economic Evaluation of Diagnostic Technologies: Comparing *Helicobacter Pylori* Screening Strategies in Prevention of Gastric Cancer in Canada,” Journal of the American College of Radiology 6, no. 5 (2009): 317–323. 19394572 10.1016/j.jacr.2009.01.022

[21] T. Leivo , A. Salomaa , T. U. Kosunen , et al., “Cost–Benefit Analysis of *Helicobacter pylori* Screening,” Health Policy 70, no. 1 (2004): 85–96. 15312711 10.1016/j.healthpol.2004.02.004

[22] Y.‐C. Lee , J.‐T. Lin , H.‐M. Wu , et al., “Cost‐Effectiveness Analysis Between Primary and Secondary Preventive Strategies for Gastric Cancer,” Cancer Epidemiology, Biomarkers & Prevention 16, no. 5 (2007): 875–885. 10.1158/1055-9965.EPI-06-0758 17507609

[23] J. Parsonnet , R. A. Harris , H. M. Hack , and D. K. Owens , “Modelling Cost‐Effectiveness of *Helicobacter Pylori* Screening to Prevent Gastric Cancer: A Mandate for Clinical Trials,” Lancet 348, no. 9021 (1996): 150–154. 8684154 10.1016/s0140-6736(96)01501-2

[24] P. Roderick , R. Davies , J. Raftery , et al., “Cost‐Effectiveness of Population Screening for *Helicobacter pylori* in Preventing Gastric Cancer and Peptic Ulcer Disease, Using Simulation,” Journal of Medical Screening 10, no. 3 (2003): 148–156. 14561268 10.1177/096914130301000310

[25] D. Mülder , J. F. O'Mahony , D. Sun , et al., “The Optimal Age of *Helicobacter Pylori* Screen‐and‐Treat for Gastric Cancer Prevention in the United States,” Helicobacter 30 (2025): e70039. 40329483 10.1111/hel.70039 PMC12056297

[26] T.‐H. Chiang , W.‐J. Chang , S. L.‐S. Chen , et al., “Mass Eradication of *Helicobacter Pylori* to Reduce Gastric Cancer Incidence and Mortality: A Long‐Term Cohort Study on Matsu Islands,” Gut 70, no. 2 (2021): 243–250. 32792335 10.1136/gutjnl-2020-322200 PMC7815911

[27] A. M. Fendrick , M. E. Chernew , R. A. Hirth , B. S. Bloom , R. R. Bandekar , and J. M. Scheiman , “Clinical and Economic Effects of Population‐Based *Helicobacter pylori* Screening to Prevent Gastric Cancer,” Archives of Internal Medicine 159, no. 2 (1999): 142–148. 9927096 10.1001/archinte.159.2.142

[28] R. A. Harris , D. K. Owens , H. Witherell , and J. Parsonnet , “Helicobacter Pylori and Gastric Cancer: What Are the Benefits of Screening Only for the CagA Phenotype of *H. Pylori*?,” Helicobacter 4, no. 2 (1999): 69–76. 10382118 10.1046/j.1523-5378.1999.98057.x

[29] S. Z. Ding , Y. Q. Du , H. Lu , et al., “Chinese Consensus Report on Family‐Based Helicobacter Pylori Infection Control and Management (2021 Edition),” Gut 71, no. 2 (2022): 238–253. 34836916 10.1136/gutjnl-2021-325630 PMC8762011

[30] W. D. Chey , C. W. Howden , S. F. Moss , et al., “ACG Clinical Guideline: Treatment of *Helicobacter Pylori* Infection,” American Journal of Gastroenterology 119, no. 9 (2024): 1730–1753. 39626064 10.14309/ajg.0000000000002968

[31] J.‐M. Liou , P. Malfertheiner , Y.‐C. Lee , et al., “Screening and Eradication of *Helicobacter pylori* for Gastric Cancer Prevention: The Taipei Global Consensus,” Gut 69, no. 12 (2020): 2093–2112. 33004546 10.1136/gutjnl-2020-322368

[32] W. K. Leung , T. L. Ang , S. Shah , et al., “Asian Pacific Association of Gastroenterology Task Force Recommendations on Surveillance for *Helicobacter Pylori* Associated Gastric Premalignant Conditions,” Gut 74 (2025): 335823. 10.1136/gutjnl-2025-335823 PMC13018803 41057234

[33] N. Shafiq , A. K. Pandey , S. Malhotra , et al., “Shortage of Essential Antimicrobials: A Major Challenge to Global Health Security,” BMJ Global Health 6, no. 11 (2021): 006961. 10.1136/bmjgh-2021-006961 PMC8565534 34728479

[34] E. Toes‐Zoutendijk , M. E. van Leerdam , E. Dekker , et al., “Real‐Time Monitoring of Results During First Year of Dutch Colorectal Cancer Screening Program and Optimization by Altering Fecal Immunochemical Test Cut‐Off Levels,” Gastroenterology 152, no. 4 (2017): 767–775.e2. 27890769 10.1053/j.gastro.2016.11.022

[35] F. Van Hees , A. G. Zauber , H. Van Veldhuizen , et al., “The Value of Models in Informing Resource Allocation in Colorectal Cancer Screening: The Case of the Netherlands,” Gut 64, no. 12 (2015): 1985–1997. 26063755 10.1136/gutjnl-2015-309316 PMC4672636

[36] S. Bansal , V. Deshpande , X. Zhao , et al., “Analysis of Mammography Screening Schedules Under Varying Resource Constraints for Planning Breast Cancer Control Programs in Low‐And Middle‐Income Countries: A Mathematical Study,” Medical Decision Making 40, no. 3 (2020): 364–378. 32160823 10.1177/0272989X20910724

[37] R. Cookson , A. J. Mirelman , S. Griffin , et al., “Using Cost‐Effectiveness Analysis to Address Health Equity Concerns,” Value in Health 20, no. 2 (2017): 206–212. 28237196 10.1016/j.jval.2016.11.027 PMC5340318

[38] A. L. V. Avanceña and L. A. Prosser , “Examining Equity Effects of Health Interventions in Cost‐Effectiveness Analysis: A Systematic Review,” Value in Health 24, no. 1 (2021): 136–143. 33431148 10.1016/j.jval.2020.10.010

[39] M. Asaria , S. Griffin , and R. Cookson , “Distributional Cost‐Effectiveness Analysis: A Tutorial,” Medical Decision Making 36, no. 1 (2016): 8–19. 25908564 10.1177/0272989X15583266 PMC4853814

[40] S. Verguet and D. T. Jamison , Health Policy Analysis: Applications of Extended Cost‐Effectiveness Analysis Methodology in Disease Control Priorities, 3rd ed. (Europe PMC, 2018). 30212165

[41] S. Verguet , Z. D. Olson , J. B. Babigumira , et al., “Health Gains and Financial Risk Protection Afforded by Public Financing of Selected Interventions in Ethiopia: An Extended Cost‐Effectiveness Analysis,” Lancet Global Health 3, no. 5 (2015): e288–e296. 25889470 10.1016/S2214-109X(14)70346-8

[42] A. M. Teng , G. Kvizhinadze , N. Nair , M. McLeod , N. Wilson , and T. Blakely , “A Screening Program to Test and Treat for *Helicobacter Pylori* Infection: Cost‐Utility Analysis by Age, Sex and Ethnicity,” BMC Infectious Diseases 17, no. 1 (2017): 156. 28219322 10.1186/s12879-017-2259-2 PMC5319166

[43] Y.‐C. Lee , H.‐M. Chiu , T.‐H. Chiang , et al., “Accuracy of Faecal Occult Blood Test and *Helicobacter pylori* Stool Antigen Test for Detection of Upper Gastrointestinal Lesions,” BMJ Open 3, no. 10 (2013): e003989. 10.1136/bmjopen-2013-003989 PMC3816242 24176798

[44] S. A. V. Nieuwenburg , M. C. Mommersteeg , E. L. Eikenboom , et al., “Factors Associated With the Progression of Gastric Intestinal Metaplasia: A Multicenter, Prospective Cohort Study,” Endosc Int Open 9, no. 3 (2021): E297–E305. 33655025 10.1055/a-1314-6626 PMC7892268

[45] Y. C. Lee , T. H. Chiang , H. M. Chiu , et al., “Community‐Based Gastric Cancer Screening Coupled With a National Colorectal Cancer Screening Program: Baseline Results,” Gastroenterology 160, no. 6 (2021): 2159–2161.e4. 33444571 10.1053/j.gastro.2021.01.008

[46] A. Shaukat and T. R. Levin , “Current and Future Colorectal Cancer Screening Strategies,” Nature Reviews. Gastroenterology & Hepatology 19, no. 8 (2022): 521–531. 35505243 10.1038/s41575-022-00612-y PMC9063618

[47] F. Zhu , X. Zhang , P. Li , and Y. Zhu , “Effect of *Helicobacter Pylori* Eradication on Gastric Precancerous Lesions: A Systematic Review and Meta‐Analysis,” Helicobacter 28, no. 6 (2023): e13013. 37602719 10.1111/hel.13013

[48] Y.‐C. Lee , T.‐H. Chiang , H.‐M. Chiu , et al., “Screening for *Helicobacter pylori* to Prevent Gastric Cancer: A Pragmatic Randomized Clinical Trial,” Journal of the American Medical Association 332, no. 19 (2024): 1642–1651. 39348147 10.1001/jama.2024.14887 PMC11581485

[49] M. Plummer , S. Franceschi , J. Vignat , D. Forman , and C. de Martel , “Global Burden of Gastric Cancer Attributable to *Helicobacter Pylori* ,” International Journal of Cancer 136, no. 2 (2015): 487–490. 24889903 10.1002/ijc.28999

[50] C. de Martel , D. Georges , F. Bray , J. Ferlay , and G. M. Clifford , “Global Burden of Cancer Attributable to Infections in 2018: A Worldwide Incidence Analysis,” Lancet Global Health 8, no. 2 (2020): e180–e190. 31862245 10.1016/S2214-109X(19)30488-7

[51] D. D. de Bondt , E. E. L. Jansen , C. Stogios , et al., “Optimizing the Harms and Benefits of Cervical Screening in a Partially Vaccinated Population in Ontario, Canada: A Modeling Study,” Medical Decision Making 45 (2025): 332597. 10.1177/0272989X251332597 PMC12166155 40260498

[52] P. Moayyedi , Y.‐C. Lee , M. Gerhard , and F. Mégraud , “Antibiotic Stewardship for Population‐Based Helicobacter Pylori Screen‐and‐Treat Programmes, Including Testing of Cure and Monitoring of Antibiotic Resistance,” in Population‐Based Helicobacter Pylori Screen‐and‐Treat Strategies for Gastric Cancer Prevention: Guidance on Implementation (International Agency for Research on Cancer, 2025). 40601795

[53] D. T. Mülder , J. F. O'Mahony , N. Kapteijn , et al., “The Disease Burden of *Helicobacter Pylori* Beyond Gastric Cancer: Quantifying the Forgotten Potential Benefits of Mass Eradication,” Gastroenterology 170 (2025): 344–352. 41236450 10.1053/j.gastro.2025.08.015

[54] S. Wernly , G. Semmler , M. Flamm , et al., “The Association Between Helicobacter Pylori and Colorectal Neoplasia,” Medical Principles and Practice 32, no. 1 (2023): 77–85. 36580903 10.1159/000528794 PMC10267487

[55] D. S. Choi , S. I. Seo , W. G. Shin , and C. H. Park , “Risk for Colorectal Neoplasia in Patients With *Helicobacter pylori* Infection: A Systematic Review and Meta‐Analysis,” Clinical and Translational Gastroenterology 11, no. 2 (2020): e00127. 32032128 10.14309/ctg.0000000000000127 PMC7145030

[56] D. M. Pritchard , J. Bornschein , I. Beales , A. Beresniak , H. Salhi , and P. Malfertheiner , “Cost‐Effectiveness Modelling of Use of Urea Breath Test for the Management of *Helicobacter Pylori*‐Related Dyspepsia and Peptic Ulcer in the UK,” BMJ Open Gastroenterology 8, no. 1 (2021): e000685. 10.1136/bmjgast-2021-000685 PMC8268888 34244244

[57] Q. Chen , X. Liang , X. Long , L. Yu , W. Liu , and H. Lu , “Cost‐Effectiveness Analysis of Screen‐And‐Treat Strategy in Asymptomatic Chinese for Preventing *Helicobacter Pylori*‐Associated Diseases,” Helicobacter 24, no. 2 (2019): e12563. 30672082 10.1111/hel.12563

