# Supplementary material for: Optimizing Benefits‐Harms of H. pylori Screen‐and‐Treat Programs Tailored to the Regional Settings
Source: Helicobacter. 2026 Mar 4;31(2):e70111. doi: 10.1111/hel.70111 (PMC12960071; doi:10.1111/hel.70111)
Supplement: Supplementary file 1 — Data S1: Supporting Information. [file HEL-31-e70111-s001.docx]

# Supplementary material

**Search query:**

(

("Stomach Neoplasms"[Mesh] OR gastric cancer[tiab] OR gastric tumour[tiab] OR gastric tumor[tiab])

AND

("Helicobacter pylori"[Mesh] OR Helicobacter pylori[tiab] OR H. pylori[tiab])

AND

("Mass Screening"[Mesh] OR screening[tiab])

AND

(

"Costs and Cost Analysis"[Mesh]

OR "Economic Evaluation"[tiab]

OR cost-effectiveness[tiab]

OR cost benefit[tiab]

OR cost-benefit[tiab]

OR economic*[tiab]

)

)
